# Impact of health beliefs, social support and self-efficacy on physical activity and dietary habits during the post-partum period after gestational diabetes mellitus: study protocol

**DOI:** 10.1186/1471-2393-13-133

**Published:** 2013-06-21

**Authors:** Barbara Kaiser, Chantal Razurel, Emilien Jeannot

**Affiliations:** 1 University of Applied Sciences Western Switzerland, Haute Ecole de Santé, 47 Avenue de Champel, 1206 Geneva, Switzerland

**Keywords:** Gestational diabetes mellitus, Type 2 diabetes prevention, Dietary habits, Physical activity, Postpartum period, Health beliefs, Social support, Self-efficacy

## Abstract

**Background:**

Gestational diabetes mellitus (GDM) is defined as a glucose intolerance of variable severity occurring or diagnosed for the first time during pregnancy. Numerous epidemiological studies show that this disorder affects between 1 and 18% of pregnancies, depending on the ethnicity of the populations studied, the diagnostic criteria, or the body mass index (BMI). Its incidence is constantly rising worldwide. Patients with GDM have a high risk of developing type 2 diabetes in the months after delivery. For this reason, GDM patients are encouraged to practice specific health behaviors (dietary habits, physical activity) during the postpartum period. It is important to identify the factors that may impact adherence to these behaviors.

**Methods/Design:**

A targeted sample size of 200 eligible pregnant women with a diagnosis of GDM will be enrolled in this prospective, cohort study. They will be recruited from 30-36 weeks of gestation as part of their diabetes consultation in Geneva University Hospital (GUH) maternity unit. Psychosocial variables that could impact adherence to health behaviors in the postpartum period (behavioral intentions, risk perceptions, general knowledge about diabetes, health beliefs, social support, self-efficacy) will be evaluated using specific tools at the end of pregnancy, at 6 weeks postpartum and at 6 months postpartum. Multiple regression analyses will be performed on SPSS.

**Discussion:**

For the first time in Europe, the objective of this research is to study in women with very recent GDM the link between dietary habits, physical activity levels, and psychosocial and cognitive factors possibly involved in the adoption of health behaviors in the postpartum period. These factors have been identified in the literature, but to date have never been combined in a single study. The study will allow a predictive theoretical model of health behavior to be established and used as a basis for reflection to optimize interventions carried out on women who have had GDM.

## Background

GDM is defined as a glucose intolerance of variable severity occurring or diagnosed for the first time during pregnancy [1]. Numerous epidemiological studies show that this disorder currently affects between 1 and 18% of pregnancies [2,3]. Its incidence is constantly rising worldwide [4]. GDM is a condition that to a large extent generates the interest of researchers and health practitioners in obstetrics since it is associated with pathological pregnancy outcomes, particularly for the newborn: macrosomia, shoulder dystocia and its corollaries, brachial plexus and collarbone fractures. With neonatal hypoglycemia, these are the most common complications of GDM [5]. Meanwhile, the diagnosis of GDM indicates that the mother has a predisposition to diabetes. According to Getahun et al., when gestational diabetes is diagnosed during a first pregnancy, women have a 41% risk of developing new GDM during a subsequent pregnancy [6]. The risk of developing type 2 diabetes is 2 to 7 times higher in women who have had GDM compared to women who have never had this condition [7,8] and a diagnosis of GDM doubles the risk of type 2 diabetes occurring in the 4 months following pregnancy [8]. These women at high risk should therefore undergo increased preventive surveillance and be informed about basic healthy lifestyle rules to adopt so as to prevent the onset of diabetes. These include the need for regular physical activity (i.e. moderate activity at least 30 minutes/day, 5 days a week, or strenuous exercise at least 20 minutes/day, 3 days a week). In addition, a balanced diet, including at least five servings of fruit and/or vegetables/day, and low in sugar, salt and fat should also be recommended [9].

The recommendations for lifestyle habits to adopt after GDM are therefore defined. Overall, dietary habits and physical activity levels rarely meet the recommendations; women with a history of GDM reported difficulties exercising and maintaining a healthy diet during the postpartum period (for a review see [10]).

One explanation for this phenomenon could be poor knowledge linked to poor understanding or interpretation of information given during pregnancy during which GDM occurs. To this end, in the context of a small qualitative study, Kapustin (2004) interviewed 5 women 2 to 3 years after the onset of GDM [11]. They relate that because they were told that diabetes disappeared after the birth of the baby, they reverted to their dietary habits after birth without applying the advice in terms of diet and physical activity provided during pregnancy.

It also appears that women who have had GDM do not perceive themselves to be at risk of developing type 2 diabetes, even though at the same time they recognize that GDM is a risk factor for type 2 diabetes [12,13]. However, as postulated by the Health Belief Model [14], a greater perception of risk is associated with a greater intention to adapt their lifestyle, especially in terms of diet and physical activity.

Thus, the concept of risk perception and, more generally, beliefs about health and health behaviors (i.e., benefits, barriers, social influences) may be significant predictors of behavioral intentions firstly, then effective health behaviors in the postpartum period after GDM [10,13,15,16].

Apart from knowledge about diabetes and its means of prevention, the concept of risk perception and health beliefs, five studies focused specifically on the psychosocial conditions associated with postpartum eating behaviors and physical activity in women who had GDM [16-21]. These conditions included self- efficacy and social support. Indeed, it showed that a high level of self-efficacy and social support were key factors for the adoption of physical activity and/or adequate dietary habits (for a review see [10]).

**Figure 1:**
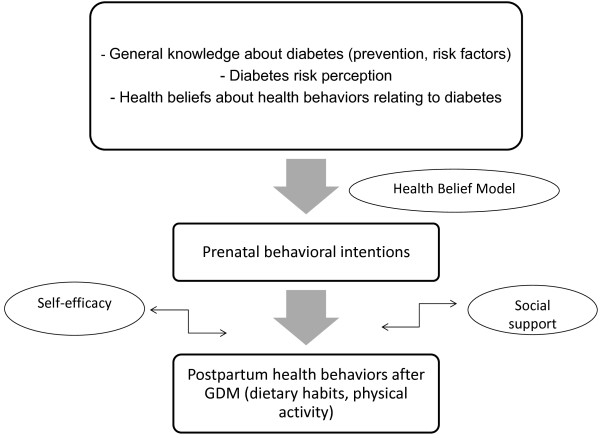
**A model of the adoption of postpartum health behaviors after GDM**[10]**.**

A number of factors are therefore likely at play in the adoption or not of adapted preventive behaviors by women who have had GDM (Figure 1). However, no study to date has combined these factors within the same protocol of longitudinal research, which is nevertheless indispensable to creating and implementing specific cognitive and psychosocial interventions in women who have had GDM so as to increase their adherence to advice on diet and physical activity by reducing the dissonance between knowledge and behavior.

### Study aims

The longitudinal data collection will be carried out (i) at the end of pregnancy during which GDM was diagnosed (T1), (ii) at six weeks postpartum (T2), and (iii) at six months postpartum. This will therefore allow three aims to be achieved:

1. The evaluation at the end of pregnancy will allow for an inventory of knowledge, risk perception and behavioral intention to be established for the postpartum period in women suffering from GDM, and therefore to evaluate the impact of informational interventions carried out by health professionals on these women during pregnancy.

2. The longitudinal design of this study will allow on the one hand the measurement of the evolution over time of health behaviors in women with GDM, and on the other hand establish the link between this evolution, behavioral intentions at the end of pregnancy, the level of knowledge on the prevention of diabetic disorder, health beliefs, self-efficacy and social support.

3. From a psychometric point of view, the linking of all of the evaluated factors will help establish an explanatory, integrative, theoretical model of adherence to healthy lifestyle rules recommended after GDM, therefore part of a broader research policy of prevention of type 2 diabetes.

## Methods/Design

### Study design and setting

A prospective cohort of 200 pregnant women with a diagnosis of GDM will be recruited at the end of pregnancy as part of prenatal consultations in the GUH maternity unit, a maternity hospital, which handles an average of 4,000 deliveries annually. This study was approved by the GUH Central Commission of Ethics and Research in 2011. Written informed consent will be obtained from all study participants.

### Study population and sampling method

The study will focus on women with a diagnosis of GDM during pregnancy, recruited between 30 and 36 weeks of gestation without previous type 1 or type 2 diabetes, aged at least 18 years, and able to read, write and speak French. The study population will comprise a lot of different ethnicities in line with the make-up of the Geneva resident population. Women with a history of GDM are not excluded from the study. The argument for this is that, unlike women who suffer from diabetes type 1 or 2, they will not have received regular medical care combined with treatment that could interfere with their health behaviors.

**Figure 2:**
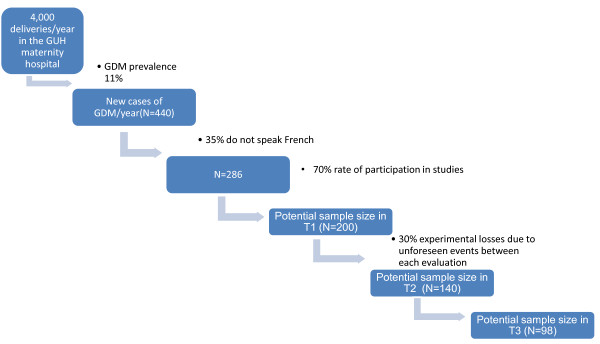
Estimation of the available sample size in T1, T2 and T3.

Since the prevalence of GDM in Geneva is 11% [22], and the GUH maternity unit handles about 4,000 deliveries per year, one can theoretically expect 440 new cases of GDM in a year. The 35% of women who do not speak French must be subtracted from this figure. Acceptance to participate in research in the population of women who have had GDM rate is estimated at 70%. The target sample size of 200 at T1 is therefore attainable (Figure 2). After taking into account up to 30% of experimental losses between each evaluation time, we estimate having a sample of 140 women in T2 and 98 women in T3.

### Sample power calculation

The Tabachnick & Fidell [23] formula was used to calculate the power of the sample size. Indeed, this formula takes into account the number of predictor variables involved in multiple regression analyzes which will be carried out in this study to construct the explanatory theoretical model of health behaviors after GDM.

For an alpha probability of .05 and a power of .80, the recommended sample size (N) to correctly evaluate the predictor variables (m) in the multivariate analyzes is: N = 50 + 8 m. In our study, six predictor variables are taken into account (knowledge about diabetes, risk perception, health beliefs, behavioral intentions, social support and self-efficacy). It will therefore be necessary to have at least 98 patients for each time. The power of the test is acceptable when the ratio “number of participants/number of predictor variables” is greater than or equal to 15, which is the case with this sample size.

### Recruitment strategies

Women will be recruited through their diabetes consultation in the GUH maternity hospital, where they go once a week for an obstetric, dietary and diabetes check-up, effective from the time of gestational diabetes diagnosis (around 24-28 weeks of gestation). Although some of these patients are followed up by independent practitioners, all women who have given birth in the GUH maternity unit have to have this consultation at the end of pregnancy, at around 36 weeks or after. Recruitment will therefore be carried out between 30 and 36 weeks of gestation.

### Study procedures

**Figure 3:**
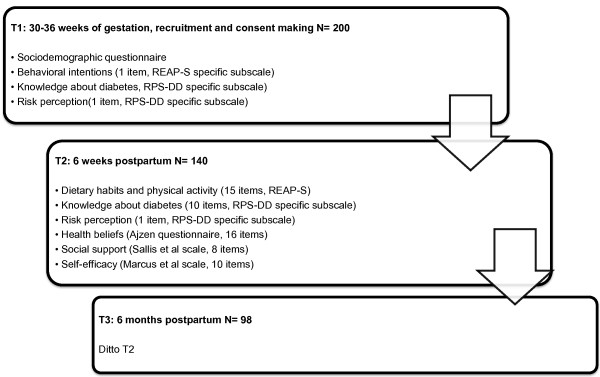
Study protocol and assessments at different time points during the study.

All of the protocol questionnaires were tested on 10 women to evaluate acceptability and understanding. No particular difficulty was identified, and the test period was estimated to be 10 minutes for T1 and 20 minutes for T2 and T3, respectively. The participants sign an informed consent form in T1 and fill out the initial questionnaires. Baseline information on socioeconomic, personal medical and obstetric history will be obtained through an interviewer-administered questionnaire. In T2 and T3, questionnaires are sent by post with a pre-stamped envelope for return. A reminder by telephone and/or by email is carried out in the event of no response Figure 3.

### Measures

#### Health behaviors: eating behaviors and physical activity

To evaluate participants’ lifestyles (dietary habits and physical activity), we will use the Rapid Eating and Activity Assessment for Participants short version (REAP-S) [24]. This is a short questionnaire (15 items, 13 for diet, 2 for physical activity) originally intended to evaluate dietary habits and physical activity in pre-diabetic patients. The questionnaire was constructed to be understandable whatever the level of education of the participants concerned. Every item corresponds to a lifestyle habit, the participant has to answer the question, “in a normal week, how many times do you…?”. The possible answers go from “Usually, Often” to “Rarely, Never”.

#### Behavioral intentions

The behavioral intentions of the participants, in other words their predisposition at the end of pregnancy towards healthy behaviors in the postpartum period, will be evaluated using 1 item from the REAP-S previously cited and we have therefore adapted the wording so that it suits a population of women at the end of pregnancy: “After your delivery, to what extent are you willing to adopt health lifestyle habits (diet, physical activity) which allow you to stay in good health?”. The possible answers go from 5 (“very willing”) to 1 (“not willing”).

#### Risk perception

The perception of risk of developing diabetes in the future will be measured by an item, used in the study by Kim et al. [13], and extracted from the Risk Perception Survey for Developing Diabetes (RPS-DD; [24,25]): “In your opinion, how big a risk is there of you developing diabetes in the next 10 years?” (response scale from 1 to 4, 1 corresponding to “almost no risk” and 4 to a “maximum risk”).

#### Level of knowledge about diabetes risk factors and preventive strategies

We will use the corresponding subscale (10 items) of the RPS-DD previously cited and for which the psychometric qualities have been highlighted [25,26]. Each of the 10 items corresponds to a diabetes risk factor for the general population. The participants have to tick for each item one of 4 possible responses: “increases risk”, “has no effect on risk”, “reduces risk”, or “don’t know”.

#### Beliefs

In agreement with the theory of planned behavior [27], beliefs about health behaviors, which are the subject of our study (physical activity and healthy diet), will be evaluated using 16 questions (8 for each target health behavior) constructed according to Ajzen’s guideline [28]. This questionnaire evaluates the perceived advantages of behaviors (3 items per target behavior, therefore 6 items), the normative influence, i.e. people who have the most influence on whether the person adopts or does not adopt these behaviors (3 items per target behavior, therefore 6 items) and the perceived barriers that prevent these behaviors from being adopted (2 items per target behavior, therefore 4 items). The questions are open of the nature, “According to you, what are the main advantages to partaking in regular physical activity?” All of the health beliefs thus identified qualitatively will be used to establish a list of the most common beliefs about physical activity and diet in a population of European women with postpartum gestational diabetes.

#### Social support

The evaluation tool we will use is specific to social support for physical activity and healthy eating. It is a revised version of the scale developed by Sallis et al. [29] to which we have added items relating to assistance participants can benefit from in terms of child care and household chores. These factors have in fact been identified as having a major influence, particularly on physical activity in women [30]. Smith et al. [17] used this tool to evaluate social support for physical activity in women in the postpartum period after gestational diabetes, highlighting good internal consistency in this population (Cronbach’s alpha = .73). Similarly, Zehle et al. [20] showed that in the postpartum period in women who have had gestational diabetes, the main impact factors on the adoption or non-adoption of a healthy diet were support in household chores, child care, preparation of meals, as well as the rest of the family’s dietary habits. We have therefore integrated additional items into the scale allowing for the evaluation of specific forms of social support in our study population. An overall score will then be obtained adding the participants’ responses to the 8 items in the scale (4 for physical activity, 4 for diet).

#### Self-efficacy

The feeling of self-efficacy for physical activity and adopting a healthy diet will be measured using a modified version of the scale developed by Marcus et al. [31], which has good-retest reliability [16]. Participants must say to what degree they feel capable (“very capable” to “not at all capable”) of partaking in physical activity and adopting a healthy diet, in 5 different situations: when they feel tired, when they are in a bad mood, when they have little time, when they are on holidays, and finally, when they find this requires too much effort. The principal component analysis revealed a satisfactory internal consistency for this scale (Cronbach’s alpha = .65; [16]), which contains 10 items (5 for physical activity, 5 for diet).

### Statistical methods

The data will be analyzed using descriptive statistics, correlational analyses, a Student’s *t* test for paired groups, discriminant and multiple regression analyses. The descriptive statistics (means, standard deviations, frequencies, percentage of sample) will be used to identify the demographic characteristics of the sample as well as to calculate the scores of participants in each of the evaluation tools. At each postpartum time, the women will be categorized according to their health behaviors. The groups then formed will be compared using *χ*^2^ tests for categorical variables and one-factor analysis of variance for ordinal variables. Transversally, the Pearson correlations will be used to examine the link between health behaviors and the dependent variables at each evaluation time, and also to evaluate longitudinally the link between behavioral intentions, risk perception, beliefs and knowledge at the end of pregnancy with the adoption of health behaviors in the postpartum period. The Student’s t tests will then evaluate the stability of variables between each evaluation time (between T1 and T2, T2 and T3, T1 and T3). The discriminant analyses (t tests), for their part, will be used to identify at each time the factors that contribute to adopting health behaviors, in other words those which discriminate women who adopt adapted behaviors.

To establish a predictive theoretical model of adoption of adapted health behaviors in the postpartum period after GDM, multiple regression analyses will be carried out. Significant variables will be used in the model in the event of a P value of the estimated beta equaling 0.05. All of the analyses will be carried out using SPSS software with a significance threshold set at 0.05.

## Discussion

For the first time in Europe, this study aims to describe the patterns of dietary behavior and physical activity in women with GDM, their intentions to modify or not modify their behaviors and their level of knowledge relating to diabetes and its prevention. Using a longitudinal protocol, the study will then observe the congruence between the behavioral intentions at the end of pregnancy, the effective adoption of health behaviors in the postpartum period after GDM, the beliefs relating to risk perception for oneself and for others, the advantages, the obstacles and the social influences attributed to behavior, and finally the concepts of self-efficacy and social support.

These results will give rise to an explanatory theoretical model of eating behaviors and physical activity in the postpartum period after GDM, a model specific to a European Genevese population characterized by great ethnic, cultural, economic and social diversity, which is not the case in the majority of existing studies on the subject [10]. A whole field of research on adapted interventions that can be proposed to patients for the prevention of type 2 diabetes and the recurrence of gestational diabetes may thus arise from this study.

Indeed, not only will the results provide multidisciplinary care teams working with GDM patients with the basis necessary to reflect on how to improve diabetes-related prevention efforts: these results could lead to nurse and midwife training in order to implement specific interventions in maternity care units. In particular, interventions based on various cognitive and behavioral strategies derived from Social Cognitive Theory (SCT) could potentially be effective for increasing health behaviors among postpartum women after a GDM. According to SCT, there are multiple influences on behavior, including both cognitive and social factors [32]. Behavioral strategies based on SCT include increasing self-efficacy, social support (eliciting support from family and friends), enjoyment of the behavior, and outcome expectancies, which refer to the degree to which the individual believes behaviors will lead to a particular outcome. It could be a telephone-based intervention, because non-face-to-face interventions may be ideal due to time constraints, child-care conflicts, and transportation constraints sometimes present for the population of postpartum women [33]. These interventions could maximize the impact of the advice given and improve the compliance to health recommendations if needed.

## Abbreviations

GDM: Gestational diabetes mellitus; BMI: Body mass index; GUH: Geneva University Hospital; T1: Evaluation at time 1, at the end of pregnancy; T2: Evaluation at 6 weeks postpartum; T3: Evaluation at 6 months postpartum

## Competing interests

The authors have no competing interest to report.

## Authors’ contributions

BK and CR led the conceptualization and design of the original project, developed this current protocol, prepared the proposal and obtained funding. EJ prepared this manuscript with input from BK. All authors reviewed and approved the final version of the manuscript.

## Authors’ information

BK: Professor of Midwifery, midwife, psychologist, PhD in Clinical Psychology.

CR: Professor of Midwifery, midwife, MSc Sciences of Education.

EJ: Professor Assistant, MSc Epidemiology.

## Pre-publication history

The pre-publication history for this paper can be accessed here:

http://www.biomedcentral.com/1471-2393/13/133/prepub
